# The Aberrant Right Subclavian Artery (Arteria Lusoria): The Morphological and Clinical Aspects of One of the Most Important Variations—A Systematic Study of 141 Reports

**DOI:** 10.1155/2014/292734

**Published:** 2014-07-01

**Authors:** Michał Polguj, Łukasz Chrzanowski, Jarosław D. Kasprzak, Ludomir Stefańczyk, Mirosław Topol, Agata Majos

**Affiliations:** ^1^Department of Angiology, Medical University of Łódź, Narutowicza 60, 90-136 Łódź, Poland; ^2^Department of Cardiology, Medical University of Łódź, Kniaziewicza 33, 90-153 Łódź, Poland; ^3^Department of Radiology, Medical University of Łódź, Kopcińskiego 22, 90-153 Łódź, Poland; ^4^Department of Normal and Clinical Anatomy, Medical University of Łódź, Narutowicza 60, 90-136 Łódź, Poland; ^5^Department of Radiological and Isotopic Diagnosis and Therapy, Medical University of Łódź, Żeromskiego 113, 90-549 Łódź, Poland

## Abstract

The most important abnormality of the aortic arch is arguably the presence of an aberrant right subclavian artery (arteria lusoria). If this vessel compresses the adjacent structures, several symptoms may be produced. The aim of the study is to present the morphological and clinical aspects of the aberrant right subclavian artery. Three different databases searched for a review of pertinent literature using strictly predetermined criteria. Of 141 cases, 15 were cadaveric and 126 were clinically documented. The gender distribution of the subjects was 55.3% female and 44.7% male. The mean age of the patients at symptoms onset was 49.9 ± 19.4 years for all patients but 54.0 ± 19.6 years and 44.9 ± 18.1 years for female and male subjects, respectively (*P* = 0.0061). The most common symptoms in this group were dysphagia (71.2%), dyspnea (18.7%), retrosternal pain (17.0%), cough (7.6%), and weight loss (5.9%). The vascular anomalies coexisting with an arteria lusoria were truncus bicaroticus (19.2%), Kommerell's diverticulum (14.9%), aneurysm of the artery itself (12.8%), and a right sided aortic arch (9.2%). In conclusion, compression of adjacent structures by an aberrant right subclavian artery needs to be differentiated from other conditions presenting dysphagia, dyspnea, retrosternal pain, cough, and weight loss.

## 1. Introduction

The most common embryologic abnormality of the aortic arch is aberrant right subclavian artery (ARSA), known clinically as arteria lusoria (AL) [1]. The first description of this variation was provided in 1735 by Hunauld [2]. However, the clinical entity of “dysphagia lusoria” was first described by Bayford in 1787 in a woman with long history of dysphagia who was found to have an aberrant right subclavian artery at autopsy [3]. Hence, it is also known as Bayford-Autenrieth dysphagia.

**Figure 1:**
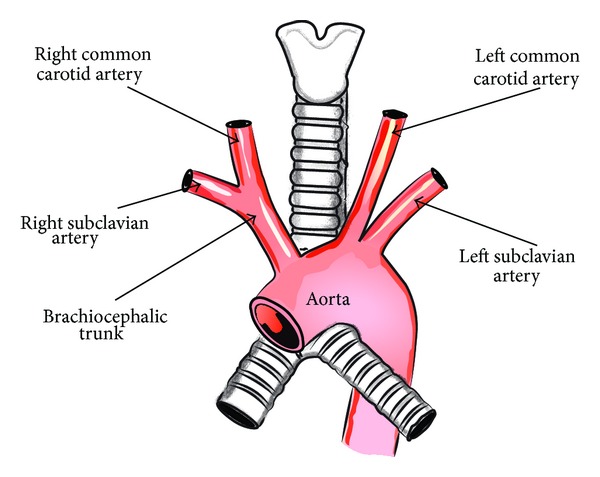
Schematic arrangements of the more common morphological variations of branches of the aortic arch.

**Figure 2:**
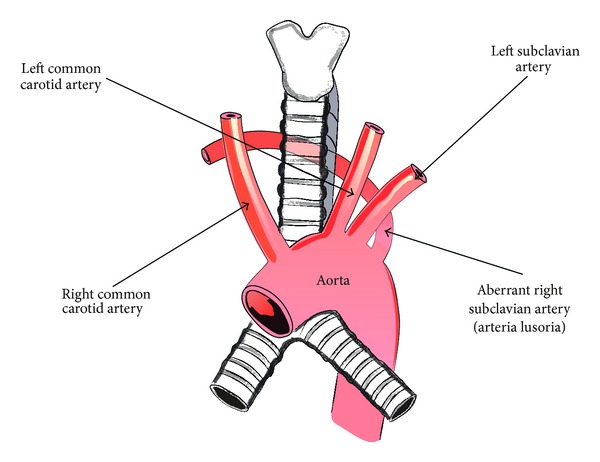
Schematic arrangements of the presence of an aberrant right subclavian artery.

Usually, three large arteries arise from the arch of the aorta: the brachiocephalic trunk (divided into the right common carotid artery and the right subclavian artery), the left common carotid artery, and the left subclavian artery [2] (Figure 1). However, when aberrant right subclavian artery variant is present, the brachiocephalic trunk is absent and four large arteries arise from the arch of the aorta: the right common carotid artery, the left common carotid artery, the left subclavian artery, and the final one with the most distal left sided origin, the right subclavian artery, also called the arteria lusoria (Figure 2). This vessel travels to the right arm, crossing the middle line of the body and usually passing behind the esophagus. If the artery compresses the esophagus, it may produce a condition called dysphagia lusoria [3, 4]. Frequently, the arteria lusoria arises from an aortic arch diverticulum at the proximal descending aorta, first described by Kommerell [4].

Normally, the right subclavian artery develops from the distal fusion of a persistent right proximal dorsal aorta with the right seventh intersegmental artery. The aberrant origin of the right subclavian artery is caused by the involution of the right fourth vascular arch and proximal right dorsal aorta and the persistence of the seventh intersegmental artery originating from the proximal descending thoracic aorta, forming the abnormal course of the artery lusoria [5, 6].

The aim of the study was to review incidences of the aberrant right subclavian artery (arteria lusoria) published between 1988 and 2013. This paper offers objective description of clinical and morphological features related to this anomaly. As “dysphagia lusoria” is an independent nosologic entity that should be differentiated from other causes of dysphagia, the findings of the present study describing the aberrant right subclavian artery are important and useful for clinicians involved in many medical fields.

## 2. Materials and Methods

### 2.1. Literature Search Procedures

A systematic review was performed in order to analyse the clinical and morphological aspects of arteria lusoria. Relevant studies were identified by searching in the following data sources: MEDLINE via PUBMED, EBSCO, and SCOPUS. Search terms included the key words “aberrant right subclavian artery” and “arteria lusoria.” Studies were eligible for inclusion in the meta-analysis if they met all of the following criteria: (1) published in the English language from 1988 to 2013, (2) reported postmortem or radiologically confirmed anomaly cases with an explicit and detailed description of the symptoms onset, and (3) contained information about gender and age of the patients at onset of symptoms. Original and review articles with summarized descriptions of cases were excluded. The coexistence of concomitant vascular anomalies was recorded.

### 2.2. Statistical Analysis

Data analysis was performed using Statistica 10 software (StatSoft Polska, Cracow, Poland). The Shapiro-Wilk's test was used to confirm whether the distribution of continuous variables was normal. Mean, median, and standard deviation (SD), as well as minimum and maximum for continuous variables, were presented as descriptive statistics. Statistical analysis was performed by employing the Mann-Whitney test. A *P* value less than 0.05 was considered significant.

## 3. Results

### 3.1. Literature Search and Selected Studies

The literature search yielded 796 abstracts from three different databases, of which 219 publications were selected to be reviewed in full text. Of these, 119 studies that did not meet our inclusion criteria were rejected. Of 100 selected studies (141 cases), 9 were cadaveric studies (15 cases) and 91 were clinically documented (126 cases). For the present meta-analysis, the three most commonly affiliated countries were the United States (24 cases), The Netherlands (20 cases), and Germany (16 cases). The geographic distribution of included cases is presented in Table 1.

### 3.2. Demographic, Clinical, and Morphological Characteristics

According to our study, the gender distribution was 55.3% (78/141) females versus 44.7% (63/141) males. The mean age of the onset of symptoms was 49.9 ± 19.4 years for the whole group (data shown as mean ± standard deviation). However, the mean age according to gender was 44.9 ± 18.1 years for males and 54.0 ± 19.6 years for females (Table 2). According to the Mann-Whitney test, the difference was statistically significant, with *P* = 0.0061.

The most commonly reported symptoms related to compression of adjacent structures by aberrant right subclavian artery (arteria lusoria) were dysphagia (71.2%), dyspnea (18.7%), retrosternal pain (17.0%), cough (7.6%), and weight loss greater than 10 kg over a 6-month period (5.9%). Among the less common symptoms, stomach-ache, back pain, and numbness of the right upper limb were reported.

The most common vascular anomalies coexisting with an aberrant right subclavian artery (arteria lusoria) were truncus bicaroticus, 19.2% (27/141); Kommerell's diverticulum, 14.9% (21/141); aneurysm (just after the origin of arteria lusoria), 12.8% (18/141); and right-sided aortic arch, 9.2% (13/141).

## 4. Discussion

The occurrence of arteria lusoria in our analysis was more common in female than male subjects (55.3% versus 44.7%), which is similar to the results given by Molz and Burri [1], who note that this anomaly was found more often in females (58%) than males (42%). Jain et al. [7] also report that the aberrant right subclavian artery has a female predominance.

According to a current bibliography search, the symptoms of arteria lusoria compression have been found to be present only in 7–10% of adult patients with the anomaly. So the anomaly is clinically silent in 90–93% of cases [8].

On the base of the review of 295 patients with ARSA Klinkhamer [9] concluded that symptoms occurred only when a common carotid trunk (truncus bicaroticus) or two very closely arising carotid arteries were presented. The study reports that the presence of AL was combined with truncus bicaroticus in 85 of 295 patients (29%) [9]. According to our study, the coexistence of truncus bicaroticus with AL was lower and was present in 19.2% (27/141) of the studied population. However, Hartyánszky et al. [10] reported the presence of only 8 symptomatic infants with an aberrant RSA with truncus bicaroticus among 111 paediatric patients (7.2%).

A 1936 study by Kommerell [4] describes a diverticulum at the origin of the aberrant right subclavian artery. This root of the arteria lusoria has a broad base and is formed by a persisting right aortic arch [1]. Clinically, it is known as Kommerell's diverticulum. Epstein and DeBord [11] noted that 60% of ARSA coexists with a Kommerell's diverticulum. It is a much higher value than seen in the present analysis (14.9%).

Kieffer et al. [12] reported the presence of an ARSA aneurysm in 10 of 33 patients (30.3%). This differs from the results of the present analysis, which notes the presence of an ARSA aneurysm in only 18 of 141 cases (12.8%) of the studied population. However, no clear distinction exists between the aneurysm and diverticulum of the ARSA, and several authors use the terms interchangeably, which might account for the differences in the results.

On the other hand, the prevalence of vascular anomalies coexisting with ARSA may depend on the sample population, as does the presence of arteria lusoria. The frequency of ARSA varies throughout the world. In Europe, depending on the country, it was found in 0.11% (Great Britain; Kelly 2007 [13]), 0.16% (Greece; Natsis et al. [14]), 0.3% (France; Abhaichand et al. [15]), or 0.38% (Nederland; de Luca et al. [16]) of the population. Studies have also been performed on other continents: Asia, 0.1-0.2% of cases (Korea; Nie et al. [17] and Japan; Saito et al. [18], resp.); North America, 0.5% of cases (United State; Haesemeyer and Gavant [19]); or Australia and Oceania, 0.8% of cases (New Zealand, Cainey [20]). However, in our opinion, its detection mainly depends on the sensitivity of diagnostic procedures, for example, cadaveric or CT versus chest X-ray examination [14, 19, 20].

The presence of an aberrant right subclavian artery is also higher in disorders such as Down's, DiGeorge, and Edwards' syndromes whole instead of all population [21]. According to Nakajima et al. [22] the incidence of the aberrant subclavian artery was 6% in patients with the tetralogy of Fallot and 16% with its combination and pulmonary atresia or major aorticopulmonary collateral arteries. Also, de Luca et al. [16] described that 6 from 12 diagnosed patients with ARSA also had Down's syndrome, ventricular septal defect, and tetralogy of Fallot.

Furthermore, some recent experimental studies have shown that abnormalities in the walls of arteries derived from the fourth arch (the proximal part of the right subclavian artery and segment B of the aortic arch) may explain why these arteries are the subject of specific anomalies and pathologies [23, 24]. Such vascular abnormalities may be a risk factor for the development of an ostial stenotic lesion of the ARSA [25]. Also, Schneider et al. [26] note that with regard to the pathophysiological mechanisms involved, aortic tear or dissection may be more likely to occur during deceleration trauma when an aberrant right subclavian artery (lusoria) is present.

Puri et al. [27] stated that symptoms, when present, occur at the two extremes of life. In children, tracheal obstruction or dysphagia can occur. The increased frequency of pulmonary infections seen in infants is thought to be due to the absence of tracheal rigidity. Van Son et al. [28] found that 86% of infant patients with AL had symptoms of stridor or recurrent respiratory infections. In infants, the trachea is compressible; therefore, the typical signs and symptoms compression by arteria lusoria are respiratory, such as wheezing, stridor, recurrent pneumonia, and cyanosis. In adults, the trachea is more rigid, and so, respiratory symptoms are rare. In adults, a congenital vascular anomaly of the aortic arch and major branches is a cause of dysphagia, classically termed as “dysphagia lusoria” [27]. Also, McNally and Rak [29] attribute this rarity to the greater likelihood of esophageal compression in adults due to tracheal rigidity. Ulger et al. [30] stated that dysphagia generally develops in older patients due to increased rigidity of the oesophagus itself or the vessel wall, elongation of the aorta, or formation of an aneurysm.

The mean age of all patients evaluated in this retrospective study was 49.9 years. However, statistically significant differences were found between the mean ages of the female and male subjects: 54.0 years versus 44.9 years, respectively. These results are in agreement with those of Levitt and Richter [31] which present the average age of symptomatic patients with ARSA as 48 years.

It is unknown why most patients having dysphagia symptoms are middle aged or older. According to a current bibliography search, various mechanisms have been proposed as to why dysphagia occurs in elderly patients: (1) increased rigidity of trachea leading to easy compression of esophagus [9], (2) aneurysm formation, especially in presence of Kommerell's diverticulum [31, 32], (3) elongation of the aorta [33], (4) increased rigidity of AL by atherosclerotic changes in the wall, and (5) the coexistence of an ARSA with a truncus bicaroticus [9, 32] or the presence of a close origin of carotid arteries from the aorta, which limits the anterior displacement of trachea and esophagus [30, 34].

According to analyzed data observations, the most explanatory concept is that connected with atherosclerosis, which is supported by symptoms occurrence in older age and also by the significant difference between the average ages of female and male patients. The atherosclerotic changes in the walls of the arteries accelerate due to reduced estrogen protection in female subjects after approximately 60–65 years of life, which corresponds to the mean age of the female subjects in our study (54.0 years). These results are also supported by several cases describing atherosclerotic AL [25, 33, 35, 36].

According to Yang et al. [37] diagnostic sensitivity in detection of aberrant right subclavian artery of 64 multislice computed tomography and Doppler sonography were 100% and 97.6%, respectively. It was also supported by Chen et al. [38] studies. Scientists stated that diagnostic sensitivity of 64 multidetector computed tomography and transthoracic echocardiography for congenital aortic anomalies were 100% and 92%, respectively [38]. However, Branscom and Austin [39] noted that abnormalities of the aortic arch in a chest X-ray picture were found in only 20% of cases with an arteria lusoria.

The first successful open surgical repair of an aberrant right subclavian artery was performed by Gross in 1946 on four-month-old infant [40]. Since this report many techniques for division of the ARSA and reconstruction of blood flow to the right arm have been described [6, 12, 25, 41]. Also a variety of surgical approaches with many different vascular reconstruction methods have been proposed [28, 42, 43]. Conventional surgical or endovascular treatment of symptomatic ARSA in the literature is still discussed extensively [6, 35, 41, 44]. Nowadays, progress of endovascular techniques have led to hybrid option with combined endovascular and open surgical repair. The hybrid method allows exclusion of the artery with thoracic endograft following occlusion of the ARSA via endovascular embolization techniques [43, 45].

A familiarity with the anatomy of the most common types of vascular anomalies is necessary for clinicians involved in many medical areas. In patients with ARSA and prolonged nasogastric or endotracheal intubation, gastrointestinal bleeding should raise suspicion of a fistulous connection [7]. Desvant et al. [46] assert that an aberrant right subclavian artery should be considered as a potential risk factor of tracheotomy bleeding. The presence of ARSA may result in unforeseen problems in transradial coronary procedures [17]. According to the literature, only 60% of procedures using a transradial approach were successful in the presence of an arteria lusoria [47]. This congenital variant makes it difficult to approach the ascending aorta by the right transradial route, as it requires the catheter to curve back to reach the aortic root [48]. The presence of ARSA together with an absence of the right recurrent laryngeal nerve is clinically important; during thyroid surgery, the right laryngeal nerve cannot be found at the lower pole of the thyroid, and it may be injured by the surgeon if it is not identified in the aberrant area or found lateral to the thyroid [49, 50].

## 5. Conclusion

It is important to remember that common conditions such as shortness of breath, retrosternal pain, cough, and weight loss may be symptoms of the compression of adjacent structures by the arteria lusoria. The presence of the ARSA, especially, should be taken into consideration to distinguish from other causes of dysphagia. The hypothesis that clinically overt arteria lusoria is due to atherosclerotic wall and vessel stiffness is supported by older age of symptomatic patients.

## Figures and Tables

**Table 1:** Geographic distribution of arteria lusoria cases.

Country	Number of described cases
United State	24
Netherlands	20
Germany	16
Japan	10
India and Turkey	7
China, Poland, and Republic of South Africa	5
Spain	4
Austria, Belgium, Czech Republic, and Great Britain	3
Canada, Greece, Israel, Italy, Malaysia, and Portugal	2
Belgium, Saudi Arabia, Chile, Durban, France, Qatar, South Korea, Liban, Oman, Pakistan, Serbia, Slovakia, Switzerland, and Tunisia	1

**Table 2:** The age distribution of the onset of symptoms associated with an aberrant right subclavian artery.

	Mean	Standard deviation	Minimum	Maximum	Median
All group	49.9	19.4	7	94	49
Female	54.0	19.6	11	94	55.5
Male	44.9	18.1	7	87	44
